# MRI-based machine learning reveals proteasome subunit PSMB8-mediated malignant glioma phenotypes through activating TGFBR1/2-SMAD2/3 axis

**DOI:** 10.1186/s43556-025-00268-5

**Published:** 2025-05-08

**Authors:** Dongling Pei, Zeyu Ma, Yuning Qiu, Minkai Wang, Zilong Wang, Xianzhi Liu, Long Zhang, Zhenyu Zhang, Ran Li, Dongming Yan

**Affiliations:** 1 Department of Neurosurgery, The First Affiliated Hospital of Zhengzhou University, Zhengzhou, 450001 Henan China; 2 MOE Laboratory of Biosystems Homeostasis & Protection and Innovation Center for Cell Signaling Network, Life Sciences Institute, Zhejiang University, Hangzhou, 310058 China; 3 School of Medicine, Hangzhou City University, Hangzhou, 310015 Zhejiang China

**Keywords:** Glioma, Proteasome subunit beta type-8 (PSMB8), Proliferation, Apoptosis, Radiomics

## Abstract

Gliomas are the most prevalent and aggressive neoplasms of the central nervous system, representing a major challenge for effective treatment and patient prognosis. This study identifies the proteasome subunit beta type-8 (PSMB8/LMP7) as a promising prognostic biomarker for glioma. Using a multiparametric radiomic model derived from preoperative magnetic resonance imaging (MRI), we accurately predicted PSMB8 expression levels. Notably, radiomic prediction of poor prognosis was highly consistent with elevated PSMB8 expression. Our findings demonstrate that PSMB8 depletion not only suppressed glioma cell proliferation and migration but also induced apoptosis via activation of the transforming growth factor beta (TGF-β) signaling pathway. This was supported by downregulation of key receptors (TGFBR1 and TGFBR2). Furthermore, interference with PSMB8 expression impaired phosphorylation and nuclear translocation of SMAD2/3, critical mediators of TGF-β signaling. Consequently, these molecular alterations resulted in reduced tumor progression and enhanced sensitivity to temozolomide (TMZ), a standard chemotherapeutic agent. Overall, our findings highlight PSMB8's pivotal role in glioma pathophysiology and its potential as a prognostic marker. This study also demonstrates the clinical utility of MRI radiomics for preoperative risk stratification and pre-diagnosis. Targeted inhibition of PSMB8 may represent a therapeutic strategy to overcome TMZ resistance and improve glioma patient outcomes.

**Supplementary Information:**

The online version contains supplementary material available at 10.1186/s43556-025-00268-5.

## Introduction

Gliomas, which account for over 80% of malignant brain tumors, are the most prevalent primary CNS tumors, with prominent recurrence propensity posing significant clinical challenges [1]. According to the World Health Organization (WHO) classification, the majority of gliomas are diffuse and stratified into three molecular subtypes: (1) Isocitrate dehydrogenase (IDH) mutant-1p/19q co-deleted (oligodendroglioma), (2) IDH-mutant-1p/19q retained (astrocytoma), and (3) IDH-wildtype glioblastoma (GBM) [2]. Although significant progress has been made in therapeutic modalities including surgical resection, radiation therapy, and systemic chemotherapy, the median overall survival period of patients with GBM remains less than 16 months [3]. In recent years, immunotherapy has come to the fore as a potentially effective treatment method for patients suffering from malignant tumors, including glioma [4, 5]. However, only a subset of glioma patients responds to immunotherapy. GBM, the most aggressive subtype, demonstrates extreme invasiveness and poor prognosis, with a mere 5.6% 5-year survival rate post-diagnosis [2, 6].

The 26S proteasome consists of the 20S proteasome, 19S (PA700) and 11S (PA28) [7]. The 20S proteasome represents the catalytic core of this enzymatic system, comprising two α-rings and two β-rings (α₁₋₇β₁₋₇β₁₋₇α₁₋₇), with each ring comprising seven subunits. The external α-rings play a critical role in controlling substrate entry into the internal catalytic domain, which is constituted by β-subunits [8]. When the PA700 and PA28 regulatory complexes associate with the α-rings of the core particle, they give rise to the 26S proteasome and immunoproteasome, respectively. Notably, the 20S proteasome is recognized for its ability to degrade oxidized proteins, a process that operates independently of ubiquitination [7]. The proteasome mediates the degradation of ubiquitinated proteins, enabling rapid intracellular turnover and generating the primary source of peptide antigens for major histocompatibility complex class I (MHC-I) presentation [9]. Proteasome subunit beta type-8 (PSMB8/LMP7), a catalytic β subunit (β5i) of the 20S proteasome, is transcriptionally upregulated by interferon-gamma (IFN-γ) [10]. A recent analysis showed that high PSMB8 methylation is associated with an inhibitory response to immunotherapy [11]. A previous study demonstrated that PSMB8 optimized the antigen presentation of MHC-I-restricted epitopes to CD8^+^ T cells by cleaving proteins after their basic and hydrophobic residues [12]. Moreover, during the development of colitis-associated colonic cancer, the pro-tumoral capacity of immunoproteasomes appears to contribute to carcinogenesis by triggering the upregulation of diverse pro-inflammatory factors, like CXCL1, CXCL2, CXCL3, IL17 A, IL1β, and IL6 [13, 14]. In gastric cancer, the high nuclear expression level of PSMB8 has been linked to the depth of tumor invasion, lymph node metastasis, and an unfavorable prognosis [15]. However, overexpression of PSMB8 increased cytotoxicity and apoptosis, and promoted radiation-induced tumor necrosis in rectal cancer [16]. Previous research in glioma demonstrated that PSMB8 promotes tumor cell proliferation and migration by downregulating cyclin family proteins, N-cadherin, and vimentin, while upregulating E-cadherin via activation of the ERK1/2 and PI3K/AKT signaling pathways [17]. Moreover, another study indicated that in glioblastoma, high expression of PSMB8 could increase tumor angiogenesis by enhancing the expression of vascular endothelial growth factor (VEGF)-A, vascular endothelial growth factor receptor (VEGFR), and CD31 [18].

Transforming growth factor beta (TGF-β), a multifunctional polypeptide cytokine, has emerged as a promising therapeutic target due to its role in regulating cell growth and differentiation [19]. The mouse models have been utilized to explore the functional significance of the TGF-β pathway in mammary gland physiology and carcinogenesis, and these studies are achieved by making adjustments to the principal components of this signaling cascade [20]. Cancer cells manipulate multiple pathways triggered by TGF-β for their own advantage, transforming TGF-β into an oncogenic factor. This factor then promotes angiogenesis, enables cancer cell invasion, leads to immunosuppression, and facilitates the self-renewal of cancer-initiating cells [21]. The cellular response to TGF-β is triggered when ligands (TGF-β1, TGF-β2, or TGF-β3) bind to TGFBR2, a serine-threonine kinase. Subsequently, TGFBR2 phosphorylates and activates TGFBR1, the primary mediator of downstream signaling transduction [22]. TGFBR1 then phosphorylates SMAD2/3, activating these transcription factors to drive TGF-β-dependent gene expression [22].

In the current research, we explored the biological function of PSMB8 during the proliferation, migration and apoptosis processes of glioma cells. We further investigated whether the elevated expression of PSMB8 has a connection with the activation of the TGF-β pathway, thereby accelerating glioma progression. Moreover, radiomics was used to preoperatively predict PSMB8 expression and it highlighted that PSMB8 is a prognostic marker and treatment target for temozolomide (TMZ)-resistant glioma.

## Results

### Image prediction and regulating mechanism for selected genes

**Fig. 1:**
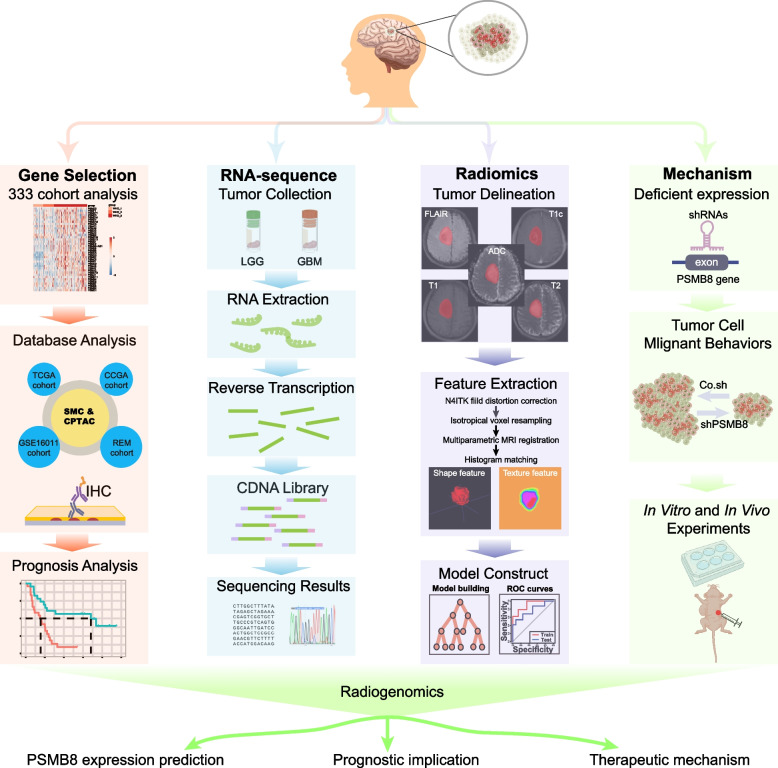
The workflow of this study. LGG: Low-grade glioma; GBM: Glioblastoma multiforme; TCGA: The Cancer Genome Atlas; CGGA: the Chinese Glioma Genome Atlas; CPTAC: Clinical Proteomic Tumor Analysis Consortium; SMC: Samsung Medical Center

Figure 1 shows an overview of the radiomics analysis. To begin with, for the purpose of normalizing the intensity and geometry, all MRI images underwent preprocessing. Specifically, the bias field distortion was rectified by making use of the N4ITK algorithm. Trilinear interpolation was used to resample isotropic voxels into 1×1×1 mm^3^ voxels. Subsequently, MRI images were rigidly registered using the axial resampled T1c images as a reference. This registration process was based on a mutual information similarity metric, ultimately generating the registered images. To accomplish intensity normalization, histogram matching was employed. The workflow included: (1) tumor delineation, (2) image preprocessing, (3) radiomics feature extraction, (4) feature selection, and (5) model construction and assessment. This research emphasized the utility of MRI radiomics in preoperative risk stratification and pre-diagnosis for targeting genes. We used our own cohort of 333 glioma cases to analyze the genes that may play crucial regulatory roles during the progression of glioma. For the selected genes, we then investigated the regulating mechanism using bioinformatics analysis and biological experimental techniques *in vitro* and *in vivo*.

### Increased PSMB8 expression in GBM was associated with poor clinical outcome

**Fig. 2:**
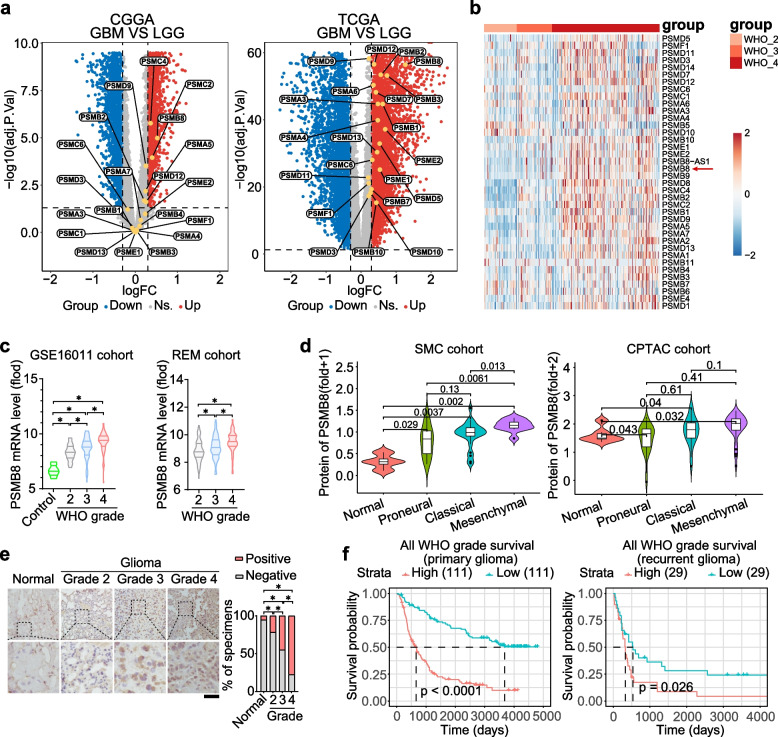
Increased PSMB8 expression in GBM was associated with poor clinical outcome. **a** Volcano plots acquired from the CGGA and TCGA data analysis, displaying the expression of the 26S proteasome subunits genes among LGG and GBM. Ns., not significant; FC, fold change. **b** A significant upregulation of PSMB8 with increasing WHO grade was determined based on mRNA-sequencing of our 333 gliomas cohort. **c** PSMB8 mRNA expression profiles in different-grade glioblastoma patients were collected from the GSE16011 and REM cohorts to compare PSMB8 expression in glioblastoma (WHO grade 4), low-grade glioma (WHO grades 2 and 3), and normal brain tissues (Control). **d** The protein levels of PSMB8 between three mRNA subtypes and normal brain tissues were detected via the MS/MC proteomic data from SMC cohort, and CPTAC cohort. **e** Left: Representative Immunohistochemistry (IHC) images of PSMB8 expression in our 110 glioma and 30 normal tissue arrays. Scar bar = 100 µm. Right: The frequency distribution of PSMB8 expression in low-stage glioma tissues (WHO grade 2, *n* = 30. WHO grade 3, *n* = 45), high-grade glioblastoma (WHO grade 4, *n* = 35), and normal brain tissues (*n* = 30). **f** Kaplan–Meier plot of overall survival of glioma patients with low and high PSMB8 expression from CGGA primary and recurrent glioma cohort. *p < 0.05. Data are analyzed of three independent experiments and presented as mean ± SD (**c**, **d**, and **e** right). Statistical analyses are performed using two-tailed Student’s *t*-test (**c**, **d**, and **e** right) and log-rank test (**f**)

Recent investigations have revealed that the abnormal gene expression of the 26S proteasome subunits family has been described in various oncogenic situations [23–26]. The volcano plot analysis of TCGA cohort (https://www.cancer.gov/ccg/research/genome-sequencing/tcga) and CGGA cohort (https://www.cgga.org.cn/) showed that PSMB8 was the most up-regulated in GBM, compared to LGG (Fig. 2a). A heat map of proteasome-related genes expression from our pre-existing mRNA sequencing dataset of 333 glioma tissues [27–29] demonstrated that the expression level of PSMB8 in WHO grade 4 gliomas was significantly elevated in contrast to that in lower-grade gliomas (WHO grades 2 and 3) (Fig. 2b), which was in line with the outcome of PSMB8 mRNA expression in the GSE16011 cohort (https://www.ncbi.nlm.nih.gov/geo/query/acc.cgi?acc=GSE16011) and REM cohort (https://www.cancerimagingarchive.net/collection/rembrandt/) (Fig. 2c). PSMB8 mRNA expression in 32 tumor types was compared using the Gene Expression Profiling Interactive Analysis database (http://gepia.cancer-pku.cn/). GBM and LGG were among the top six tumor types expressing PSMB8 (Fig. S1a). To gain insight into PSMB8 at the proteomic level, we investigated global proteomic data from the Samsung Medical Center cohort [30] and Clinical Proteomic Tumor Analysis Consortium protein dataset [31]. PSMB8 protein expression profiles in patients of different subtypes showed that PSMB8 was more highly expressed in tumor tissues. Moreover, patients with the malignant mesenchymal subtype and the highest PSMB8 had a worse prognosis than those in the other two subtypes (Fig. 2d). To further investigate PSMB8 expression in gliomas, we explored its expression level in gliomas and normal brain tissues using immunohistochemistry. Analysis of 110 glioma and 30 normal brain tissue arrays indicated that PSMB8 levels were significantly higher in tumor tissues, and elevated PSMB8 protein expression was associated with higher grades (Fig. 2e). Additionally, analysis of pre-existing RNA sequencing data sourced from the CGGA revealed that there was a negative correlation between PSMB8 and prognosis in both primary and recurrent gliomas (Fig. 2f).

### Development of the multiparametric radiomic model based on preoperative MRI

All 162 patients with information on the expression of PSMB8 were separated into a training set (*n* = 121) and a validation set (*n* = 41). And the training set (age, 52.4 ± 13.8 years) included 82 (67.8%) high expression, and 39 (32.2%) low expression PSMB8 gliomas (cutoff value = 52.48). Concurrently, in the validation set (age, 49.9 ± 11.6 years), there were 34 (82.9%) high PSMB8 expression and 7 (17.1%) low PSMB8 expression gliomas. Clinical factors, including sex (*P* = 1.00), age (*P* = 0.92), WHO grade (*P* = 0.56) and PSMB8 expression (*P* = 0.10) between the training and validation sets were not significantly different (*P* < 0.05, considered statistically significant), as shown in Supplementary Table S1.

**Fig. 3:**
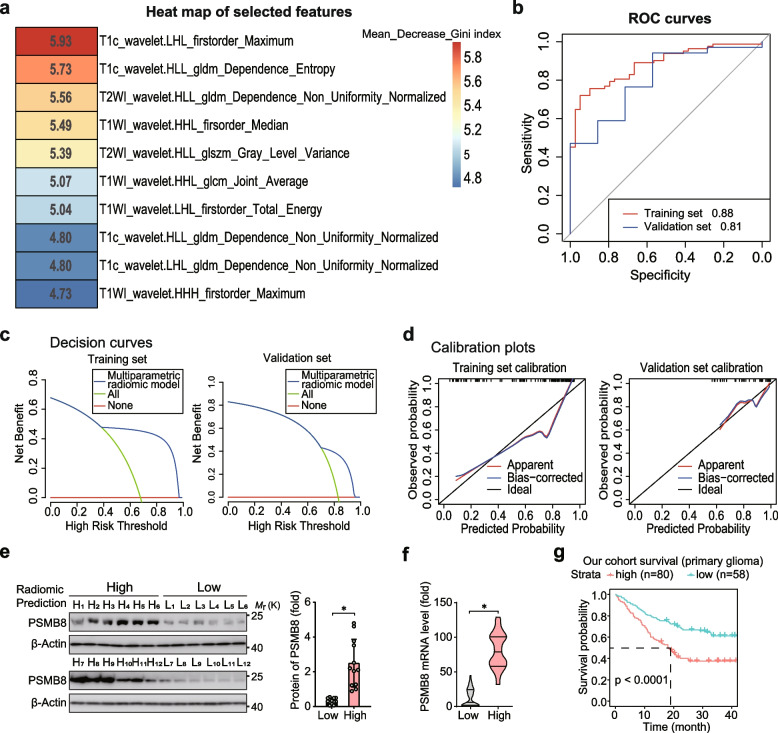
A graphic illustration of classification performance of the multiparametric radiomic model for the expression of PSMB8. **a** The heat map of the univariate contribution of each 10 selected features for the prediction of the expression of PSMB8; a higher Mean-Decrease-Gini index value indicated better classification performance of a feature. **b** ROC curves of both the training and validation sets; AUC values are shown in this part. **c** The decision curves for the prediction of the expression of PSMB8; the multiparametric radiomic model showed a great overall net benefit in most ranges of the threshold probabilities. **d** The calibration curve of the multiparametric radiomic model; there was a high agreement between prediction and observation. **e** Left: Immunoblot (IB) of the protein expression levels of PSMB8 in normal tissues and glioma tissues with different WHO grades. Right: Quantitative analysis of the expression levels (relative band density compared to β-actin) in normal tissues (Normal) and glioma tissues with different WHO grades (grades 2, 3, and 4). **f** RT-qPCR analysis of normal tissues and glioma tissues with different WHO grades. **g** The overall survival analysis of another 138 patients with glioma in terms of radiomics. *p < 0.05. Data are analyzed of at least three independent experiments and presented as mean ± SD (**e** right and **f**). Statistical analyses are performed using two-tailed Student’s *t*-test (**e** right, and **f**) and log-rank test (**g**)

For each patient, a quantity of 5929 radiomic features were extracted out of multiparametric MRI, including T1-weighted (T1), T2-weighted (T2), fluid-attenuated inversion recovery (FLAIR), T1-weighted gadolinium contrast-enhanced (T1c), diffusion-weighted (DWI) imaging, and apparent diffusion coefficient (ADC) maps. Subsequently, 1402 features remained after the stage of intraclass correlation coefficient (ICC) and redundancy reduction. Heat maps representing the correlation coefficients of all nonredundant feature sets are shown in Fig. S2a. Ten highly relevant radiomic features, including six texture features and four intensity features, were selected after Boruta selection (Fig. S2b, Supplementary Table S2. A significant correlation was found between the 10 selected features and the expression level of PSMB8 (false discovery rate-adjusted *P* < 0.01). The classification performance of the multiparametric radiomic model, which was used to predict the expression of PSMB8, in the training and validation sets is presented in Supplementary Table S3. The univariate contribution of each of the ten selected features for the expression of PSMB8 prediction can be described using the Mean-Decrease-Gini index. A heat map of the Mean-Decrease-Gini index is shown in Fig. 3a. Figure 3b displays the receiver operating characteristic (ROC) curves for both the training set and the validation set. The area under the curve (AUC) values of these two sets were 0.88 and 0.81, respectively. And the decision curves for predicting the expression of PSMB8 are shown in Fig. 3c, which indicates a substantial overall net benefit throughout the majority of the ranges of threshold probabilities. Simultaneously, the calibration curve of the multiparametric radiomic model showed a striking level of concordance between the predicted values and the actual observations, as shown in Fig. 3d. A greater Mean-Decrease-Gini index value indicates that a feature exhibits superior classification performance in predicting the expression of PSMB8. The detailed definitions of the 10 selected features are described in Supplementary Table S4.

Then, another 138 glioma patients with multiparametric MRI data were encompassed in the present study for validating. And the results of radiomic model showed 80 in high groups and 58 in low groups. For the samples of these 138 patients, high and low groups were subjected to IB. Compared with the low group, PSMB8 was significantly more abundantly present in the high group (Fig. 3e), consistent with the results of real-time reverse transcription PCR (RT-qPCR) (Fig. 3f). Analysis of the 138 glioma cohort sequencing data indicated that high subtype of PSMB8, predicted via radiomics was negatively correlated with prognosis, compared with low subtype (Fig. 3g). All the results showed that radiomics has potential to preoperatively predict the expression of PSMB8 and its relation with clinical prognosis (Fig. 3e-g).

### PSMB8 downregulation reduced the malignant behaviors of glioma cell lines in vitro

**Fig. 4:**
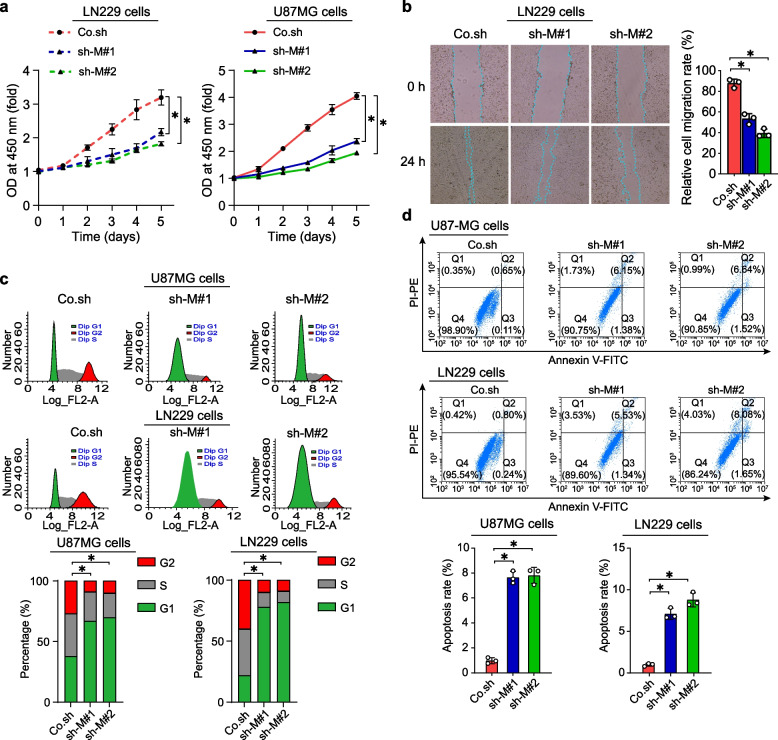
PSMB8 downregulation reduced the malignant behaviors of glioma cell lines in vitro. **a** Cell proliferation rate was evaluated in GBM cell lines after transfection using the CCK-8 assay. **b** Wound healing assay of Co.sh, sh-M #1, and sh-M #2 in LN229 cells after 24 h. **c** The effects of PSMB8 knockdown on the cell cycle were determined using flow cytometry. **d** The effects of PSMB8 knockdown on apoptosis were examined using flow cytometry. *p < 0.05. Data are analyzed of three independent experiments and presented as mean ± SD (**a**, **b** right, **c** and **d** down). Statistical analyses are performed using two-tailed Student’s *t*-test (**a**, **b** right, **c** down and **d** down)

Based on the aforementioned findings, we put forward the hypothesis that a reduction in the expression of PSMB8 could potentially impede the progression of GBM. Thus, we silenced PSMB8 in LN229 and U87 MG GBM cell lines via lentiviral infection. Transfection efficiency was evaluated using fluorescence (Fig. S3a), RT-qPCR (Fig. S3b), and IB (Fig. S3c), suggesting that PSMB8 knockdown cell models were successfully constructed in both cell lines. We then explored the effects of PSMB8 downregulation on GBM malignancy based on CCK-8, wound healing, and flow cytometric assays. PSMB8 suppression resulted in a substantial reduction in the proliferation rate, self-renewal ability, and migratory capacity of GBM cells (Fig. 4a–d). Moreover, flow cytometry analysis suggested that PSMB8 knockdown arrested the cell cycle (Fig. 4c). Simultaneously, fluorescence-activated cell sorting analysis revealed that the apoptosis rate in PSMB8-silenced GBM cells was remarkably higher compared to that in control cells (Co.sh) (Fig. 4d).

### PSMB8 expression correlates with TGF-β signaling pathway activity in glioma

**Fig. 5:**
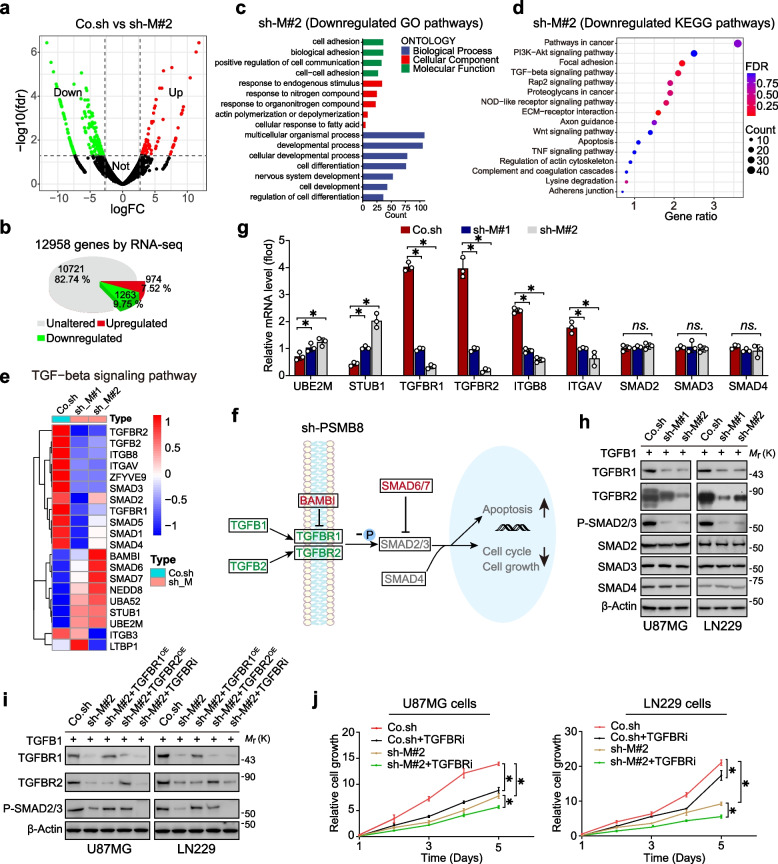
PSMB8 expression correlates with TGF-β signaling pathway activity in glioma. **a** A volcano plot illustrating differentially regulated gene expression from RNA-sequencing analysis between the Co.sh group and sh-M#1 group. Genes upregulated and downregulated in the sh-M#1 group compared with those in the control group are shown in red and green, respectively. **b** RNA-seq comparison of Co.sh and sh-M#2 revealed a total of 12,958 genes expressed, of which 974 genes were upregulated and 1263 genes were downregulated. **c** Gene ontology functional clustering of genes that were downregulated in biological processes. **d** KEGG pathway analysis of genes that were downregulated in PSMB8-deficient transcriptome. **e** Heatmap of representative TGF-β signaling pathway-related genes. **f** TGF-β signaling pathway in KEGG; The red or blue targets represent the upregulated or downregulated KEGG targets, respectively. **(g, h)** The expression of several TGF-β signaling pathway-related genes identified by RT-qPCR **(g)** and IB **(h)**. **i** TGFBRi treatment or TGFBR1/2 overexpression (OE) in PSMB8-silenced cells and immunoblot of the phosphorylation of SMAD2/3, TGFBR1, and TGFBR2. **j** Growth of PSMB8-silenced cells treated with or without TGFBRi. *p < 0.05; *ns.*, not significant. Data are analyzed of three independent experiments and presented as mean ± SD (**g**, **j**). Statistical analyses are performed using two-tailed Student’s *t*-test (**g**, **j**)

To uncover the mechanism by which PSMB8 facilitates malignant glioma cell behavior, we applied RNA sequencing analysis to Co.sh, sh-M#1, and sh-M#2 LN229 cells to determine transcriptional changes. Among the significantly differentially regulated genes (DRGs), transcripts of 2319 (sh-M#1, 17.8%) and 1263 (sh-M#2, 9.75%) genes were downregulated, whereas transcripts of 1958 (sh-M#1, 15.03%) and 974 (sh-M#2, 7.52%) were upregulated in PSMB8 knockdown cells as compared to the control LN229 cells (Fig. 5a, b, and S4a-b). Furthermore, gene ontology (GO) enrichment analysis conducted on the DRGs disclosed there were significantly impacted gene categories. These genes were either downregulated or upregulated as a response to PSMB8 deficiency. The downregulated genes of Co.sh vs sh-M#2 were associated with multicellular organismal processes, responses to endogenous stimuli, and cell adhesion (Fig. 5c, Supplementary Table S5), suggesting that PSMB8 was closely associated with cellular biological processes and migration in cancer. Upregulated GO terms indicated anatomical structure development and cellular response to chemical stimuli (Fig. S4c, Supplementary Table S5). Furthermore, KEGG pathway analysis of downregulated genes of Co.sh vs sh-M#2 showed that the genes were enriched within the ECM-receptor interaction, focal adhesion, and the TGF-β signaling pathway (Fig. 5d, Supplementary Table S6), whereas those enriched in ribosome and oxidative phosphorylation were upregulated in PSMB8-knockdown glioma cells (Fig. S4d, Supplementary Table S6). Moreover, many downregulated DEGs were crucial for the TGF-β signaling pathway (Fig. 5e). Additionally, KEGG pathway enrichment analysis of all genes selected from the differentially expressed genes showed that the TGF-β signaling pathway might exert crucial functions in glioma progression (Fig. 5f). We validated the differentially altered targets of the TGF-β pathway using RT-qPCR (Fig. 5g). In line with the RNA-seq data, RT-qPCR verified that UBE2M and STUB1 were upregulated in PSMB8-deficient cells, whereas TGFBR1, TGFBR2, ITGB8, and ITGAV were downregulated (Fig. 5g). We further used IB to determine the differentially altered protein levels of the key protein targets (Fig. 5h). The biochemical results showed that TGFBR1 and TGFBR2 levels were significantly reduced based on IB and RT-qPCR (Fig. 5g, h). Here, we combined knock down of PSMB8 with a short-term course of treatment with galunisertib (LY2157299), which is a small molecule inhibitor targeting TGFBR1 (hereafter referred to as TGFBRi) (Fig. 5i, j). After silencing PSMB8, reduced expression of TGFBR1 and TGFBR2 led to decreased phosphorylation of SMAD2/3 (Fig. 5h), which inhibited the malignant behavior of glioma cells (Fig. 5j). Moreover, TGFBRi treatment further inhibited the phosphorylation of SMAD2/3 and tumor proliferation. However, the opposite result was observed in PSMB8-silenced cells overexpressing TGFBR1 or TGFBR2 (Fig. 5i, j).

### PSMB8 suppression restrains GBM growth and enhances the sensibility of TMZ in mice

**Fig. 6:**
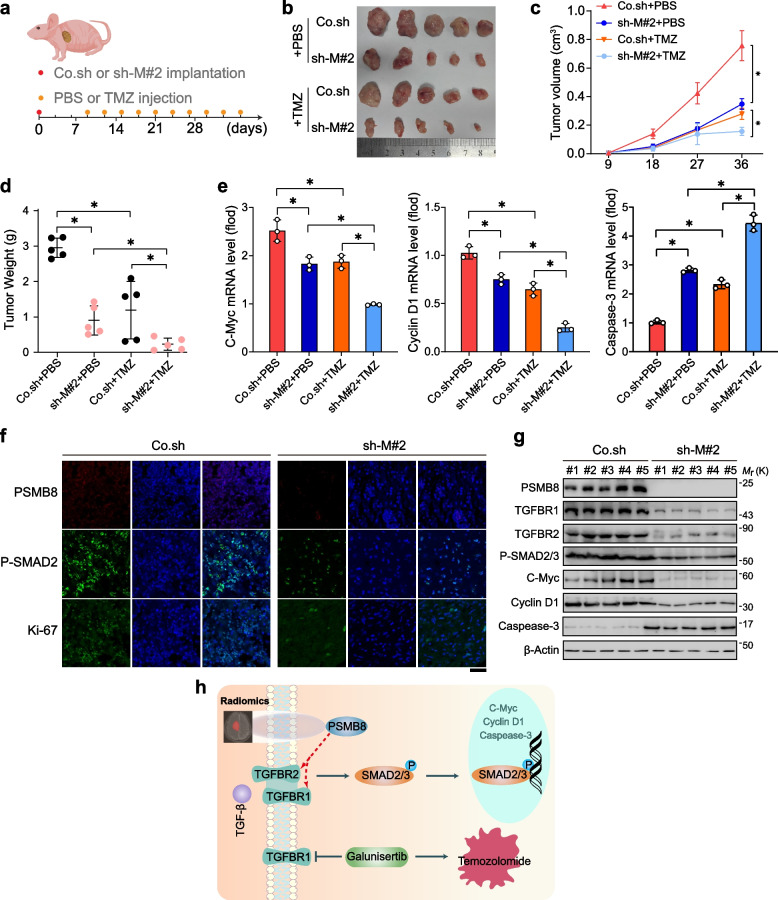
PSMB8 suppression restrains GBM growth and enhances the sensibility of TMZ in mice. **a** Diagram of the experimental procedure. U87MG cells (5 × 10^6^ cells) transfected with Co.sh and sh-M#2 were subcutaneously injected into BALB/c nude mice (*n* = 5 mice per group). Nine days after injection, mice were injected with PBS or TMZ every 3 days. **b** Representative images xenograft tumors of the nude mice. **c** Curves of tumor growth in the PSMB8 knockdown group and control group with or without TMZ treatment were analyzed. The tumor volumes were measured every 9 days. **d** Tumor weights of each group were measured after being surgically dissected. **e** RT-qPCR analysis of c-Myc, cyclin D1, and caspase-3 mRNA expression in tumors from four different treatment groups. **f** PSMB8, p-SMAD2, and Ki67 expression in tumor tissues of nude mice was determined via immunofluorescence staining. Scar bar = 100 µm. **g** Immunoblotting analysis of tumor tissues. **h** Schematic illustration of radiomic prediction of PSMB8 expression, which enhanced the efficacy of temozolomide via the TGF-β signaling pathway. *p < 0.05; Data are representative of three independent experiments (**e**) and are shown as mean ± SD. Statistical analyses were performed using a two-tailed Student’s *t*-test (**c-e**)

The development of TMZ resistance remains a major obstacle to GBM treatment [32]. To verify that the knockdown of PSMB8 was able to suppress the growth of GBM tumors and improve the effectiveness of TMZ in vivo, U87MG (5 × 10^6^) cells underwent subcutaneous injection into the armpits of 6-week-old experimental BALB/c nude mice. Subsequently, these nude mice were thereafter grouped into four groups according to PSMB8 silencing and presence or absence of TMZ treatment. Nine days after the injection, the mice underwent intraperitoneal injection treatments with TMZ or the same volume of phosphate buffer saline (PBS) every 3 days (Fig. 6a). Compared with the control group, tumors in the sh-M#2 group or the TMZ treatment group had smaller sizes, weights, and volumes. Moreover, in the group receiving TMZ treatment, PSMB8 knockdown led to an additional decrease in these parameters (Fig. 6b-d). After that, immunofluorescence staining was employed to examine the expression of PSMB8, p-SMAD2, and Ki-67 in the transplanted tumors of the Co.sh and sh-M#2 groups. The results indicated that, when compared to Co.sh-transduced xenografts, the expression magnitudes of PSMB8, p-SMAD2, and Ki-67 in the sh-M#2-transduced GBM xenografts were significantly reduced (Fig. 6f). In line with this, RT-qPCR analysis demonstrated that knockdown of PSMB8 or TMZ treatment dramatically reduced the expression of c-Myc and cyclin D1 (tumor growth markers) and enhanced caspase- 3 (tumor apoptosis marker) (Fig. 6e). Depletion of PSMB8 or TMZ combination inhibited GBM tumor growth by reducing the downstream protein expression and SMAD2 phosphorylation of the TGF-β pathway (Fig. 6g). Taken together, our results showed that PSMB8 could stabilize or enhance the expression of TGFBR1/2, and the subsequent increase in the phosphorylation of SMAD2/3 promoted nuclear translocation and downstream target genes of the TGF-β pathway (Fig. 6h).

## Discussion

Gliomas rank among the most prevalent and severe types of human cancers. The diagnosis of glioma relies on histopathological criteria and molecular genetics, and tumors are categorized into different grades in accordance with the WHO classification system. This categorization is intimately associated with the prognosis and treatment approaches for glioma patients [33]. Glioblastoma (WHO grade 4) represents the most common glioma among adults and is characterized by an unfavorable prognosis [33–35]. Even with the existing standard treatment, involving the maximum safe resection, and then radiotherapy in combination with TMZ [36], and a range of salvage therapies being available once tumor progression takes place, the vast majority of patients still succumb to the disease within two years after being diagnosed [3, 37].

PSMB8 is a major component of the immunoproteasome [38], which is found mainly in monocytes and lymphocytes and is known to process MHC-I [39]. PSMB8 also has a significant impact on myatrophy, arthrogryposis, panniculitis-associated lipodystrophy syndrome, and microcytic anemia [38]. Earlier research has indicated that PSMB8 exhibits high expression levels in tumor tissues. Moreover, its expression level rises as gliomas progress [17, 18]. In other words, the expression level of PSMB8 is significantly higher in high-grade gliomas as opposed to low-grade gliomas. Additionally, it was discovered that the ERK1/2 and PI3 K/AKT signaling pathways triggered by PSMB8 played a role in the migration and proliferation processes of glioma cells [17]. However, our study focused on uncovering the role of PSMB8-mediated malignant glioma phenotypes through the activation of the TGFBR1/2-SMAD2/3 axis. This new direction of our investigation explored a different molecular mechanism compared to the previously identified pathways, which may offer potential therapeutic targets that have not been considered before in the context of glioma treatment related to PSMB8's actions. TGF-β signaling plays important roles in inducing epithelial-mesenchymal transition, cell growth, differentiation, and morphogenesis [40–42]. Some studies have shown that TGF-β can induce migration and invasion of various cancer cell types, including gliomas [43]. Previous research also shown that aggressive and highly proliferative gliomas exhibit high TGFβ-Smad activity, resulting in a poor prognosis for patients [44]. TGF-β1, along with TGFBR1/2, shows expression only in malignant gliomas and is not expressed in normal brain tissues, gliosis, or low-grade astrocytomas [45, 46]. Our study also showed that blocking the key proteins of TGF-β signaling pathway can be a new therapeutic target.

An escalating number of investigations imply that radiomics has multiple applications in glioma, including early diagnosis and classification, as well as prediction of biological behavior and prognosis [47–49]. After incorporation of molecular pathology into the traditional histological analysis used for classifying gliomas, a steadily increasing number of studies have centered on machine-learning-based radiomics analysis with the aim of uncovering molecular markers such as IDH mutations, 1p/19q codeletion, and various other markers [50–52]. A non-invasive approach for preoperatively forecasting key molecular markers in gliomas holds clinical importance, and we combined radiomics with genomics from a novel perspective to explore the image recognition ability of new gene markers.

To date, few studies have clarified the association between PSMB8 and prognostic value prediction via MRI. In this study, we constructed a multiparametric radiomic model based on preoperative MRI, which achieved high AUC values for predicting high and low PSMB8 expression. Moreover, we investigated the downstream signaling pathway via knockdown of PSMB8 in glioma cell lines.

The radiomic model attained high AUC values for PSMB8 based on single-center data. Nevertheless, international multi-institutional datasets are required for further validation. While we showed the silenced PSMB8 could reduce the expression of TGFBR1/2, how PSMB8 regulated directedly and why PSMB8 effects the expression of them together. It required further research that the mechanism of PSMB8 enhanced the sensitivity of TMZ in GBM patients.

## Conclusion

Collectively, our study revealed that PSMB8 can not only be a useful prognostic factor in glioma patients, but also a predictive marker of prognostic factors and novel therapeutic target by blocking key proteins of TGF-β signaling.

## Materials and methods

### Development of the radiomic model

A total of 162 patients with the expression of PSMB8 were included in the current MRI-based radiomics study. The standards for patient inclusion were (1) age ≥ 18 years; (2) pathologically confirmed WHO grade 2,3,4 diffuse gliomas; (3) availability of multiparametric MRI, including T1, T2, FLAIR, T1c, and ADC; (4) each image without any artifacts, confirmed by a neurosurgeon (ZYZ); (5) absence of preoperative medication treatment, chemotherapy, and radiation therapy; and (6) availability of information on the expression of PSMB8.

MRI images were acquired using 1.5 T or 3.0 T clinical MR scanners with different imaging parameters, and the detailed information on the MR machines and imaging parameters was summarized in Supplementary Methods A, and Table S7. To standardize the intensity and geometry, all MRI images were preprocessed. The bias field distortion was rectified by making use of the N4ITK algorithm [53]. The detailed process of image normalization and the delineation of the tumors was described in Supplementary Methods B. The radiomic features were extracted using the Python tool PyRadiomics [54]. A summary of the detailed procedure for radiomic feature extraction can be found in Supplementary Methods C.

The ICC values corresponding to each feature of the 30 selected patients were calculated with R software (version 4.2.1, www.Rproject.org) to measure the repeatability. Features with ICC values higher than 0.7 were retained. Subsequently, the patients were randomly partitioned into a training set and a validation set according to a 7:3 ratio. Detailed information on the radiomic feature selection was presented in the Supplementary Methods D. The multiparametric radiomic model for classifying the expression of PSMB8 (high and low expression, cutoff value = 52.48) was built by applying a probability algorithm based on random forest. For the purpose of optimizing the multiparametric radiomic model, tenfold cross-validation was applied.

### Patients

The First Affiliated Hospital of Zhengzhou University's Human Scientific Ethics Committee approved this study (Approval No. 2019-KY-176). All the steps of procedures were implemented in conformity with the relevant guidelines and regulatory requirements set forth by the First Affiliated Hospital of Zhengzhou University. For the patients whose fresh tumor specimens were utilized for mRNA-sequencing, quantitative reverse transcription PCR, IHC, and western blotting, informed consent was duly acquired. And informed consent was also acquired from the patients who consented to undergo MRI scans.

### Cell culture and reagents

Human glioblastoma cell lines LN229 and U87MG were procured from the Cell Resource Center of Science (Shanghai, China). The cultivation of these cells took place in Dulbecco’s modified Eagle’s medium (DMEM) which had 10% FBS (Cat No. FSP500, ExCell Bio) added to it. All the cells were maintained in a humid environment at a temperature of 37℃ with a 5% carbon dioxide atmosphere. Moreover, they were confirmed to be free from mycoplasma contamination.

### Lentivirus transduction

By transfecting HEK293 T cells with plKO-1 plasmids (designed to knock down PSMB8 shRNA) along with the helper plasmids pCMV-VSVG, pMDLg-RRE (gag/pol), and pRSV-REV, lentiviruses were generated. Forty-eight hours post-transfection, the cell supernatants were collected. These supernatants were either utilized to infect cells or stored at -80°C. To establish stable cell lines, GBM cells at a low confluence level of 20% were infected for 24 h with lentiviral supernatants that were diluted 1:1 in normal culture medium. Forty-eight hours following the infection, the cells underwent puromycin selection for a duration of one week. Subsequently, they were subcultured before being used. A concentration of 1 µg/ml of puromycin was employed to sustain the selection pressure on the stably transfected GBM cells. The lentiviral shRNAs were identified and tested, and the shRNA with the highest efficacy was selected for use:

- TRCN0000007268 (sh-M#1): CCACGTTAAGTCCAAGGAGAA;
- TRCN0000007269 (sh-M#2): CCACTCACAGAGACAGCTATT;

### Cell proliferation detection

The LN229 and U87MG cells infected with shRNAs were seeded into 96-well plates at a density of 5.0 × 10^3^ cells per well. After that, the plates were placed in an incubator maintained at 37 °C with a 5% CO₂ atmosphere and cultured overnight. At 24, 48, 72, 96, and 120 h after changing the medium, the old solution was discarded. Each well received 10 μL of the CCK-8 solution, and the plates were then kept in an incubator at 37 °C for 4 h for the incubation process. The optical density (OD) value of each experimental well at a wavelength of 450 nm was measured using a microplate reader. The purpose of this measurement was to monitor the variations in the cell proliferation capacity among different groups.

### Wound healing

Initially, the cell concentration was set at 3 × 10^5^/ml and then seeded into a 6-well plate. Following incubation, once it was observed that the cells had achieved 100% confluence, a 200 µl sterile pipette tip was employed to draw three vertical lines. Subsequently, the cells that had detached were cleaned using sterile PBS. After that, 2 ml of DMEM was added to each well. Eventually, the scratches were examined under a microscope and captured at 0, and 24 h.

### Apoptosis assays

An Annexin V-fluorescein isothiocyanate (FITC)/propidium iodide (PI) Apoptosis Detection kit (Cat#C1062L, Beyotime) was used to evaluate apoptosis of LN229 and U87MG cells infected with shRNAs. The cells were gathered and rinsed on two occasions using precooled PBS. Subsequently, they were resuspended in 1 × binding buffer. After that, 5 µl of FITC and 5 µl of PI were added. After a 15-min incubation at room temperature without light, apoptosis was analyzed using a flow cytometer.

Meanwhile, precooled 70% ethanol was used to fix the cells at 4 °C throughout the night. The following day, the fixed cells underwent centrifugation and were washed once with precooled PBS. Afterward, 500 µl of the prepared PI staining buffer was gently added to each tube to fully resuspend the cells. Subsequently, the cells underwent incubation in a 37 °C warm bath in the dark for 30 min. Finally, a flow cytometer was used to measure the DNA content of the cells.

### RNA isolation and quantitative real-time PCR (RT-qPCR)

The NucleoSpin RNA II kit (BIOKE, Netherlands) was utilized to extract RNAs from the entire population of cells. Next, the process of reverse transcribing cDNA was accomplished by utilizing the RevertAid First Strand cDNA Synthesis Kit (Fermentas). Real-time PCR was conducted using a StepOnePlus real-time PCR system (Applied Bioscience 7500) with SYBR Green (Applied Bioscience). Each sample was analyzed in at least three replicates, and GAPDH functioned as an internal calibration standard. The primer sequences employed are listed in Supplementary Table S8.

### Immunoblotting

Cells were subjected to lysis using 1 ml of lysis buffer (comprising 20 mM Tris–HCl at pH 7.4, 2 mM EDTA, 25 mM NaF, and 1% Triton X-100) supplemented with protease inhibitors (Cocktail, Bimake). This lysis process was carried out at 4 °C for 10 min. After that, the samples were centrifuged at 12 × 10^3^ g for 10 min. Subsequently, the concentrations of the proteins were ascertained. Next, equivalent quantities of the proteins within the lysate were segregated via SDS–polyacrylamide gel electrophoresis (SDS–PAGE). Following SDS–PAGE, a transfer operation was carried out to move the proteins onto a nitrocellulose membrane. The membranes were first blocked in a blocking buffer (5% skim milk in TBS-T) for 1 h at room temperature. Then, immunoblotting was executed. And in this process, specific antibodies were employed, followed by the use of secondary anti-mouse or anti-rabbit antibodies that had been conjugated to horseradish peroxidase (Amersham Biosciences). Chemiluminescence was employed for visualization. The antibodies that were employed in IB procedure were as follows: PSMB8 at 1:1000 (IB; Cat#PTM-5783, PTM-biolab), β-actin at 1:20,000 (IB; Cat#AC004, Abclonal), SMAD2 at 1:1,000 (IB; Cat#A-11, Santa Cruz), SMAD3 at 1:5,000 (IB; Cat#9513, Cell Signaling), SMAD4 at 1:1,000 (IB; Cat#B8, Santa Cruz), TGFBR1 at 1:1,000 (IB; Cat#C-12, Santa Cruz), TGFBR2 at 1:1,000 (IB; Cat#E-6, Santa Cruz), and phospho-SMAD2/3 at 1:1,000 (IB; Cat#AP0548, Abclonal).

### Immunohistochemical analysis

The normal brains and glioma tissues were immersed in 4% Paraformaldehyde (PFA) for fixation, then encased in paraffin, and finally sliced into coronal sections with a thickness of 8 µm. Next, the sections underwent a process where they were first deparaffinized by being immersed in xylene. Then, they were rehydrated by passing them through a series of ethanol solutions with gradually decreasing concentrations. This was followed by an antigen retrieval step. After that, the sections were treated with 0.3% H₂O₂ for 20 min to suppress endogenous peroxidase activity. Then, they were incubated with 5% goat serum for 30 min. Next, the sections were incubated with the specified primary antibodies against PSMB8 (Cat#PTM-5783, PTM-biolab), p-SMAD2 (Cat#A-11, Santa Cruz) or Ki 67 (Cat#A20018, Abclonal) at 4 °C for 12 h. Following the incubation process, the sections underwent a washing step using PBS. Then, they were placed in an environment of 37 °C for a 60-min incubation period with biotinylated secondary antibodies. Once the sections were washed again with PBS, they were treated with diaminobenzidine (DAB) for staining and then with hematoxylin for counterstaining.

### Subcutaneous tumor model

Mice experiments were approved by the First affiliated Hospital of Zhengzhou University Animal Welfare Committee. Female BALB/c nude mice that were six weeks old were obtained from the animal husbandry center of the Shanghai Institute of Cell Biology, Academia Sinica, situated in Shanghai, China. Prior to the commencement of the experiment, these mice were maintained in a specific pathogen-free setting for a period of three days. The mice were distributed randomly into two groups, where each group contained 10 mice. Subcutaneous injections of Co.sh or shPSMB8 cells were administered into the flanks of nude mice, at a rate of 5 million (5 × 10⁶) cells per mouse. Nine days after the injection of tumor cells, Co.sh or shPSMB8 mice were randomly partitioned into two distinct groups (*n* = 5 per subgroup). For one subgroup, intraperitoneal administration of TMZ was carried out at a dose of 5 mg/kg, with the injection being administered every third day, while the other subgroup received an equal volume (100 µl) of PBS. This treatment continued until the 36 th day. After that, the mice were subjected to euthanized. The tumors were removed and weighed. Subsequently, tumors tissues were fixed with optimal cutting temperature compound (OTC) for immunofluorescence staining, and the tissues mRNA or proteins was extracted for RT-qPCR or immunoblotting.

### Statistical analysis

R software was utilized to carry out the statistical analyses. To assess the equilibrium in the distribution of clinical information such as sex, age, WHO grades, and PSMB8 expression between the training and validation sets, either the Wilcoxon test or the chi-square (× 2) test was employed. The classification capabilities of the multiparametric model were put to the test and evaluated based on metrics including accuracy, sensitivity, specificity values, and AUC value of the ROC curve. The optimal threshold of the AUC value was calculated based on the maximum Youden index (sensitivity + specificity—1). Subsequently, employing the identical threshold that was chosen from the training set, the multiparametric radiomic model underwent testing in the validation set. Unpaired two-tailed Student’s t-tests were used to examine the differences in values between two groups, while ANOVA was applied for multivariate analysis. Utilizing the Kaplan–Meier approach, survival curves were formulated, and the log-rank test was carried out to ascertain the *P* values. GraphPad Prism 7 (GraphPad Inc., La Jolla, CA, USA) was also employed for performing statistical analysis and graphing. In the Figures, *P* values were denoted by asterisks: *p < 0.05. *P* value smaller than 0.05 was considered to be statistically significant.

## Supplementary Information

Supplementary Material 1.

## Data Availability

The sequencing data generated in this study have been deposited in the Genome Sequence Archive in National Genomics Data Center, China National Center for Bioinformation/Beijing Institute of Genomics, Chinese Academy of Sciences (GSA-Human: HRA010916, https://ngdc.cncb.ac.cn/gsa-human/browse/HRA010916, and HRA006184, https://ngdc.cncb.ac.cn/gsa-human/browse/HRA006184). Other de-identified datasets that were produced and/or analyzed throughout the present study are accessible from the corresponding author in response to a legitimate and reasonable request.
