# Supplementary material for: MRI-based machine learning reveals proteasome subunit PSMB8-mediated malignant glioma phenotypes through activating TGFBR1/2-SMAD2/3 axis
Source: Mol Biomed. 2025 May 8;6:28. doi: 10.1186/s43556-025-00268-5 (PMC12058589; doi:10.1186/s43556-025-00268-5)
Supplement: Supplementary file 1 — Supplementary Material 1. [file 43556_2025_268_MOESM1_ESM.docx]

**MRI-based machine learning reveals proteasome subunit PSMB8-mediated malignant glioma phenotypes through activating TGFBR1/2-SMAD2/3 axis**

Dongling Pei^1^*, Zeyu Ma^1^*, Yuning Qiu^1^*, Minkai Wang^1^, Zilong Wang^1^, Xianzhi Liu^1^, Long Zhang^2^, Zhenyu Zhang^1 ¶^, Ran Li^3 ¶^, and Dongming Yan^1¶^

^1^Department of Neurosurgery, The First Affiliated Hospital of Zhengzhou University, Zhengzhou, Henan, China, 450001

^2^MOE Laboratory of Biosystems Homeostasis & Protection and Innovation Center for Cell Signaling Network, Life Sciences Institute, Zhejiang University, Hangzhou, China, 310058

^3^School of Medicine, Hangzhou City University, Hangzhou, Zhejiang, China, 310015

***** Dongling Pei, Zeyu Ma, and Yuning Qiu contributed equally to this work

^¶^**Corresponding authors:**

1. Dongming Yan, Department of Neurosurgery, The First Affiliated Hospital of Zhengzhou University, Zhengzhou, Henan, China, 450001, Email: [mrdmyan@163.com](mailto:(1199204142@zju.edu.cn)).
2. Ran Li, School of Medicine, Hangzhou City University, Hangzhou, Zhejiang, China, 310015, Email: ranli1993@zju.edu.cn.
3. Zhenyu Zhang, Department of Neurosurgery, The First Affiliated Hospital of Zhengzhou University, Zhengzhou, Henan, China, 450001, Email: fcczhangzy1@zzu.edu.cn.

**Supplementary Material**

**Supplementary Methods A. MRI acquisition**

**Supplementary Methods B. Image preprocessing and tumor delineation**

**Supplementary Methods C. Radiomic feature extraction**

**Supplementary Methods D. Feature selection**

**Supplementary Table S1. Characteristics of patients of this study in the training and validation sets.**

**Supplementary Table S2. 10 selected features of multiparametric radiomic model for predicting the expression of PSMB8.**

**Supplementary Table S3. Summary of the prediction performance of the multiparametric radiomic model.**

**Supplementary Table S4. Definitions of the 10 radiomic features constituting the multiparametric radiomic model.**

**Supplementary Table S5. List of the downregulated or upregulated terms of Gene Ontology (GO) pathway analysis.**

**Supplementary Table S6. List of the downregulated or upregulated terms of KEGG pathway analysis.**

**Supplementary Table S7. MR imaging parameters in First Affiliated Hospital of Zhengzhou University (FAHZZU).**

**Supplementary Table S8. Primers for RT-qPCR.**

**Supplementary Figure S1. The expression of PSMB8 in 33 cancer types and matched TCGA normal data.**

**Supplementary Figure S2. The heatmaps of the correlation coefficients of the imaging features and the result of the Boruta feature selection.**

**Supplementary Figure S3. Knockdown the expression of PSMB8 in GBM cell lines.**

**Supplementary Figure S4. PSMB8 correlates with TGF-beta signaling pathway activity in glioma cancer.**

**Supplementary Methods A.**

**MRI acquisition**

All patients in the First Affiliated Hospital of Zhengzhou University were performed on either 1.5 T or 3.0 T clinical MR scanners with different manufacturers and imaging parameters. The 1.5 T scanners included Siemens Healthcare (Magnetom Aera/Avanto, Erlangen, Germany), and Philips Healthcare (Achieva, Best, Netherlands). The 3.0 T scanners included GE Healthcare (Discovery MR750/Signa HDxt, Milwaukee, WI, USA), Siemens Healthcare (Magnetom Verio/Prisma/Trio TIM/Skyra, Erlangen, Germany), and Philips Healthcare (Ingenia, Best, Netherlands). The brain imaging protocol includes the following sequences: (1) axial T1; (2) axial T2; (3) axial FLAIR; (4) axial T1c; (5) DWI. The contrast-enhanced sequences were acquired immediately after intravenous administration of a 0.1 mmol/kg dose of gadolinium-based contrast agent, followed by a 20-ml saline flush with an injection velocity of 2.0 ml/s. DWI was acquired before injection of the contrast agent and were used a spin echo single-shot echo-planar sequence including the values of b = 0 and b = 1000 s/mm^2^, with diffusion sensitizing gradients encoded in the x, y, and z directions. The DWI and the corresponding ADC maps generated with the software incorporated into the MRI unit. Detailed information about the MR machines and imaging parameters is summarized in **Supplementary Table S7.**

**Supplementary Methods B.**

**Image preprocessing and tumor delineation**

Trilinear interpolation was used to resample isotropic voxels into 1×1×1 mm^3^ voxels. Then, using a mutual information similarity metric, the T1, T2, FLAIR images and ADC maps were rigidly registered based on the axial resampled T1c images to to yield the registered images, referred to as rT1, rT2, rFLAIR, rT1c, rADC, respectively. Contrast-enhancing, non-enhancing, and necrotic areas comprised the entire tumor region, which were considered as the three-dimensional volume of interest (VOI). Using ITK-SNAP software (version 3.4.0, [www.itk-snap.org),](http://www.itk-snap.org),) a neurosurgeon (ZYZ with more than 12 years of experience, blinded to the clinical, pathological data and the expression of PSMB8) manually drawn VOIs in the axial rFLAIR images layer by layer. Then, in order to increase the reproducibility, 30 patients were randomly selected to re-delineate VOIs by another neurosurgeon (ZYM with more than 3 years of experience). These two experts determined the controversial delineation scope.

**Supplementary Methods C.**

**Radiomic feature extraction**

First, using the VOI from the rT1, rT2, rFLAIR, rT1c and rADC images of each patient, all radiomics features were extracted including volume and shape, intensity, and texture features. Using a Laplacian of a Gaussian kernel with four sigma levels (2.0, 3.0, 4.0, and 5.0), all original images were wavelet transformed and filtered. Volume and shape features were only extracted from the original image, which described the 3D characteristics of the tumor shape. First-order intensity features were extracted from the original and transform-domain images, which described the intensity distributions of the voxel intensities. Texture features were extracted from the original and transform-domain images using the following five different matrices: the gray-level co-occurrence matrix, gray-level run length matrix, gray-level size zone matrix, gray-level dependence matrix, and neighborhood gray-tone difference matrix. Then, a total of 1197 features including 14 volume and shape features, 234 intensity features, and 949 texture features were extracted from each imaging sequence of multiparametric MRI (T1, T2, FLAIR, T1c images and ADC maps) for each patient.

**Supplementary Methods D.**

**Feature selection**

The process of the feature selection including two parts: (1) features redundancy reduction, (2) optimal all-relevant features selection using the Caret and Boruta package for R. Specifically, z-score normalization was used to standardize features of the training set to zero mean and unit variance. Then, using the same distribution, features in the validation sets were also normalized. For feature pairs in the training set with correlation coefficients higher than 0.95, the feature with the lower univariate predictive power (higher Mann-Whitney U test P-value) was removed using the Caret package. Then, based on the remaining robust and non-redundant radiomic features, the optimal all relevant features were selected and non-useful features were screened out using the Boruta package for R. After evaluating all possible feature combinations, the best radiomic features were acquired from the training set and applied to the validation set.

**Supplementary Table S1. Characteristics of patients of this study in the training and validation sets.**

| Characteristic | Overall  (n = 162) | Training  (n = 121) | Validation  (n = 41) | *P*-value |
| --- | --- | --- | --- | --- |
| Sex |  |  |  | 1.00 |
| Male | 94 (58.0%) | 70 (57.9%) | 24 (58.5%) |  |
| Female | 68 (42.0%) | 51 (42.1%) | 17 (41.5%) |  |
| Age (year)* | 51.8 (±13.3) | 52.4 (±13.8) | 49.9 (±11.6) | 0.92 |
| WHO grades |  |  |  | 0.56 |
| Grade Ⅱ | 20 (12.3%) | 15 (12.4%) | 5 (12.2%) |  |
| Grade Ⅲ | 14 (8.6%) | 10 (8.3%) | 4 (9.8%) |  |
| Grade Ⅳ | 128 (79.0%) | 96 (79.3%) | 32 (78.0%) |  |
| Expression of PSMB8 |  |  |  | 0.10 |
| High (≥52.48) | 116 (71.6%) | 82 (67.8%) | 34 (82.9%) |  |
| Low (<52.48) | 46 (28.4%) | 39 (32.2%) | 7 (17.1%) |  |

Data are numbers of patients, with percentages in parentheses.

*Data are means ± standard deviations.

**Supplementary Table S2. 10 selected features of multiparametric radiomic model for predicting the expression of PSMB8.**

| No. | Selected Features | Type | Sequence | Filter | pFDR |
| --- | --- | --- | --- | --- | --- |
| f1 | Firstorder_Maximum | Intensity | T1c | Wavelet.LHL | <0.001 |
| f2 | Gldm_DependenceEntropy | Texture | T1c | Wavelet.HLL | <0.001 |
| f3 | Gldm_DependenceNonUniformityNormalized | Texture | T2WI | Wavelet.HLL | <0.001 |
| f4 | Firstorder_Median | Intensity | T1WI | Wavelet.HHL | <0.001 |
| f5 | Glszm_GrayLevelVariance | Texture | T2WI | Wavelet.HLL | <0.001 |
| f6 | Glcm_JointAverage | Texture | T1WI | Wavelet.HHL | <0.001 |
| f7 | Firstorder_TotalEnergy | Intensity | T1WI | Wavelet.LHL | <0.001 |
| f8 | Gldm_DependenceNonUniformityNormalized | Texture | T1c | Wavelet.HLL | <0.001 |
| f9 | Gldm_DependenceNonUniformityNormalized | Texture | T1c | Wavelet.LHL | <0.001 |
| f10 | Firstorder_Maximum | Intensity | T1WI | Wavelet.HHH | <0.001 |

pFDR is short for false discovery rate-adjusted *P* value.

**Supplementary Table S3. Summary of the prediction performance of the multiparametric radiomic model.**

| Sets | ACC | SEN | SPE | AUC (95% CI) |
| --- | --- | --- | --- | --- |
| Training | 0.79 | 0.72 | 0.95 | 0.88 (0.82, 0.94) |
| Validation | 0.73 | 0.69 | 0.86 | 0.81 (0.73, 0.98) |

ACC, accuracy; SEN, sensitivity; SPE, specificity; AUC, area under curve; CI, confidence interval.

**Supplementary Table S4. Definitions of the 10 radiomic features constituting the multiparametric radiomic model.**

To fully characterize the image phenotypes within the tumor, radiomic features were extracted from not only the original medical images but also transformed, or derived images by using wavelet or Laplacian of Gaussian (LoG) filters onto the original images. Then, radiomic features were extracted from three types of images: original images, wavelet images, and LoG images. Wavelet images were obtained by applying wavelet transform on the original images. Wavelet transform can decouple informative textures by decomposing the original images into multiple low- and high-frequency components. Let *H* and *L* be a high-pass and low-pass wavelet function, respectively. Then, the eight decomposed images can be denoted as ***I****_HHH_*, ***I****_HHL_*, ***I****_HLH_*, ***I****_HLL_*, ***I****_LHH_*, ***I****_LHL_*, ***I****_LLH_*, ***I****_LLL_*, where the three subscripts meant the high- or low-pass filtering operations along *x*, *y* and *z* directions of the original 3D MR image. LoG images were obtained by applying LoG filtering operation on the original images. LoG performs two filtering operations, a Gaussian filtering, and a Laplacian filtering. Finally, 10 features comprising 6 texture features and 4 intensity features were selected for the multiparametric radiomic model building. All 10 features were shown in **Supplementary Table S2**. The definitions of these features are described as follows.

| No. | Selected Features | Definitions |
| --- | --- | --- |
| f1 | T1c_Wavelet.LHL_Firstorder_Maximum | The maximum gray level intensity within the ROI. |
| f2 | T1c_Wavelet.HLL_Gldm_DependenceEntropy | Measures the variance in dependence entropy in the image. |
| f3 | T2WI_Wavelet.HLL_Gldm_DependenceNonUni-  formityNormalized | Measures the similarity of dependence throughout the image, with a lower value indicating more homogeneity among dependencies in the image. This is the normalized version of the DLN formula. |
| f4 | T1WI_Wavelet.HHL_Firstorder_Median | The median gray level intensity within the ROI. |
| f5 | T2WI_Wavelet.HLL_Glszm_GrayLevelVariance | GLV measures the variance in gray level intensities for the zones. |
| f6 | T1WI_Wavelet.HHL_Glcm_JointAverage | Returns the mean gray level intensity of the ii distribution. |
| f7 | T1WI_Wavelet.LHL_Firstorder_TotalEnergy | Total Energy is the value of Energy feature scaled by the volume of the voxel in cubic mm. |
| f8 | T1c_Wavelet.HLL_Gldm_DependenceNonUni-  formityNormalized | Measures the similarity of dependence throughout the image, with a lower value indicating more homogeneity among dependencies in the image. This is the normalized version of the DLN formula. |
| f9 | T1c_Wavelet.LHL_Gldm_DependenceNonUni-  formityNormalized | Measures the similarity of dependence throughout the image, with a lower value indicating more homogeneity among dependencies in the image. This is the normalized version of the DLN formula. |
| f10 | T1WI_Wavelet.HHH_Firstorder_Maximum | The maximum gray level intensity within the ROI. |

**Table S5. List of the downregulated or upregulated terms of Gene Ontology (GO) pathway analysis.**

1. List of the downregulated terms of GO pathway analysis following Co.sh vs sh-M#2.

| GO accession | Term | PValue | FDR |
| --- | --- | --- | --- |
| GO:0032502 | developmental process | 3.02E-11 | 1.20E-07 |
| GO:0030154 | cell differentiation | 1.37E-09 | 7.29E-07 |
| GO:0048869 | cellular developmental process | 1.47E-09 | 7.29E-07 |
| GO:0007399 | nervous system development | 6.35E-09 | 2.79E-06 |
| GO:0032501 | multicellular organismal process | 7.39E-08 | 2.92E-05 |
| GO:0007155 | cell adhesion | 2.79E-07 | 1.00E-04 |
| GO:0022610 | biological adhesion | 3.10E-07 | 1.02E-04 |
| GO:0098609 | cell-cell adhesion | 4.59E-07 | 1.40E-04 |
| GO:0098742 | cell-cell adhesion via plasma-membrane adhesion molecules | 2.39E-06 | 5.57E-04 |
| GO:0048468 | cell development | 4.00E-06 | 7.92E-04 |
| GO:0010243 | response to organonitrogen compound | 2.94E-05 | 0.004683 |
| GO:0009719 | response to endogenous stimulus | 2.96E-05 | 0.004683 |
| GO:1901698 | response to nitrogen compound | 3.56E-05 | 0.005415 |
| GO:0045595 | regulation of cell differentiation | 4.51E-05 | 0.006297 |
| GO:0008064 | regulation of actin polymerization or depolymerization | 1.24E-04 | 0.013686 |
| GO:0010647 | positive regulation of cell communication | 1.88E-04 | 0.018208 |
| GO:0007156 | homophilic cell adhesion via plasma membrane adhesion molecules | 1.95E-04 | 0.018208 |
| GO:0071398 | cellular response to fatty acid | 2.56E-04 | 0.023029 |
| GO:0008154 | actin polymerization or depolymerization | 3.20E-04 | 0.02642 |

1. List of the upregulated terms of GO pathway analysis following Co.sh vs sh-M#2.

| ID | Term | P-Value | FDR |
| --- | --- | --- | --- |
| GO:0001944 | vasculature development | 9.69E-06 | 0.004345 |
| GO:0060429 | epithelium development | 1.40E-05 | 0.004345 |
| GO:0008219 | cell death | 1.56E-04 | 0.029645 |
| GO:0009888 | tissue development | 2.00E-04 | 0.030268 |
| GO:0001763 | morphogenesis of a branching structure | 2.20E-04 | 0.030268 |
| GO:0035295 | tube development | 2.88E-04 | 0.030268 |
| GO:0048856 | anatomical structure development | 5.48E-04 | 0.03981 |
| GO:0009719 | response to endogenous stimulus | 3.90E-05 | 0.01003 |
| GO:0070887 | cellular response to chemical stimulus | 8.05E-05 | 0.018606 |
| GO:1901701 | cellular response to oxygen-containing compound | 2.71E-04 | 0.030268 |
| GO:0048514 | blood vessel morphogenesis | 5.04E-04 | 0.03887 |
| GO:0042542 | response to hydrogen peroxide | 6.82E-04 | 0.046369 |
| GO:0045892 | negative regulation of transcription, DNA-templated | 2.32E-04 | 0.030268 |
| GO:1903507 | negative regulation of nucleic acid-templated transcription | 2.37E-04 | 0.030268 |
| GO:1902679 | negative regulation of RNA biosynthetic process | 2.67E-04 | 0.030268 |
| GO:2000377 | regulation of reactive oxygen species metabolic process | 2.85E-04 | 0.030268 |
| GO:0051253 | negative regulation of RNA metabolic process | 4.15E-04 | 0.035502 |
| GO:0000302 | response to reactive oxygen species | 4.58E-04 | 0.037566 |

**Table S6. List of the downregulated or upregulated terms of KEGG pathway analysis.**

1. List of the downregulated terms of KEGG pathway analysis following Co.sh vs sh-M#2.

| Database | Term | Count | Ratio | P-Value | FDR |
| --- | --- | --- | --- | --- | --- |
| KEGG_PATHWAY | ECM-receptor interaction | 20 | 1.6 | 2.00E-06 | 5.90E-04 |
| KEGG_PATHWAY | Focal adhesion | 27 | 2.2 | 4.10E-04 | 3.10E-02 |
| KEGG_PATHWAY | TGF-beta signaling pathway | 26 | 2.1 | 2.90E-03 | 1.50E-01 |
| KEGG_PATHWAY | NOD-like receptor signaling pathway | 22 | 1.8 | 6.50E-03 | 2.80E-01 |
| KEGG_PATHWAY | Proteoglycans in cancer | 23 | 1.9 | 1.10E-02 | 3.70E-01 |
| KEGG_PATHWAY | Rap2 signaling pathway | 23 | 1.9 | 1.40E-02 | 4.20E-01 |
| KEGG_PATHWAY | Lysine degradation | 10 | 0.8 | 1.80E-02 | 4.40E-01 |
| KEGG_PATHWAY | Axon guidance | 19 | 1.5 | 4.10E-02 | 7.80E-01 |
| KEGG_PATHWAY | Pathways in cancer | 45 | 3.6 | 4.10E-02 | 7.80E-01 |
| KEGG_PATHWAY | PI3K-Akt signaling pathway | 31 | 2.5 | 6.50E-02 | 9.50E-01 |
| KEGG_PATHWAY | Wnt signaling pathway | 17 | 1.4 | 7.50E-02 | 9.50E-01 |
| KEGG_PATHWAY | Regulation of actin cytoskeleton | 11 | 0.9 | 7.50E-02 | 9.50E-01 |
| KEGG_PATHWAY | Adherens junction | 9 | 0.7 | 8.10E-02 | 9.50E-01 |
| KEGG_PATHWAY | Complement and coagulation cascades | 10 | 0.8 | 9.00E-02 | 9.50E-01 |
| KEGG_PATHWAY | Apoptosis | 14 | 1.1 | 9.20E-02 | 9.50E-01 |
| KEGG_PATHWAY | TNF signaling pathway | 12 | 1 | 1.00E-01 | 9.50E-01 |

1. List of the upregulated terms of KEGG pathway analysis following Co.sh vs sh-M#2.

| Database | Term | Count | Ratio | P-Value | FDR |
| --- | --- | --- | --- | --- | --- |
| KEGG_PATHWAY | Ribosome | 67 | 7.2 | 2.40E-43 | 7.00E-41 |
| KEGG_PATHWAY | Oxidative phosphorylation | 45 | 4.8 | 6.40E-24 | 9.40E-22 |
| KEGG_PATHWAY | Thermogenesis | 57 | 6.1 | 8.60E-23 | 8.40E-21 |
| KEGG_PATHWAY | Retrograde endocannabinoid signaling | 25 | 2.7 | 8.50E-07 | 1.80E-05 |
| KEGG_PATHWAY | RNA polymerase | 8 | 0.9 | 1.80E-03 | 3.50E-02 |
| KEGG_PATHWAY | Pyrimidine metabolism | 10 | 1.1 | 3.30E-03 | 5.70E-02 |
| KEGG_PATHWAY | Ribosome biogenesis in eukaryotes | 14 | 1.5 | 5.10E-03 | 8.30E-02 |
| KEGG_PATHWAY | Metabolic pathways | 103 | 11 | 8.30E-03 | 1.20E-01 |
| KEGG_PATHWAY | Spliceosome | 16 | 1.7 | 1.10E-02 | 1.60E-01 |
| KEGG_PATHWAY | Nucleotide metabolism | 11 | 1.2 | 1.40E-02 | 1.90E-01 |
| KEGG_PATHWAY | Proteasome | 7 | 0.8 | 3.40E-02 | 4.40E-01 |
| KEGG_PATHWAY | Drug metabolism - other enzymes | 9 | 1 | 6.20E-02 | 7.60E-01 |
| KEGG_PATHWAY | VEGF signaling pathway | 7 | 0.8 | 9.30E-02 | 1.00E+00 |

**Supplementary Table S7. MR imaging parameters in First Affiliated Hospital of Zhengzhou University (FAHZZU)**.

| Training and validation sets from FAHZZU | | | | | | | | | | |
| --- | --- | --- | --- | --- | --- | --- | --- | --- | --- | --- |
| **MR system** | **GE Healthcare** | | **Siemens Healthcare** | | | | | | **Philips Healthcare** | |
|  | **GE Discovery MR750/MR750w** | **GE Signa HDxt** | **Siemens**  **Verio** | **Siemens**  **Prisma** | **Siemens**  **Trio TIM** | **Siemens**  **Skyra** | **Siemens**  **Aera** | **Siemens**  **Avanto** | **Philips Ingenia** | **Philips Achieva** |
| **Field strength (T)** | 3 | 3 | 3 | 3 | 3 | 3 | 1.5 | 1.5 | 3 | 1.5 |
| **T1/T1c** |  | |  | | | | | |  | |
| **TR (ms)** | 1715-3019 | | 163-280 | | | | | | 250-498 | |
| **TE (ms)** | 19-43 | | 2.46-5.21 | | | | | | 2.032-15 | |
| **TI (ms)** | 673-945 | | N/A | | | | | | N/A | |
| **Section thickness (mm)** | 5 | | 5 | | | | | | 5-6 | |
| **Image slice spacing (mm)** | 1-2 | | 1.5-2 | | | | | | 1-1.5 | |
| **FA (°)** | 90-111 | | 70-90 | | | | | | 45-75 | |
| **number of averages/excitations** | 1-2 | | 1-3 | | | | | | 1-2 | |
| **Pixel size (mm^2^)** | 0.4297×0.4297-0.4883×0.4883 | | 0.4492×0.4492-0.8984×0.8984 | | | | | | 0.4492×0.4492-0.575×0.575 | |
| **Matrix** | 320×224-288×288 | | 256×162-320×320 | | | | | | 184×184-232×232 | |
| **FOV (mm^2^)** | 240×180-240×240 | | 230×185-240×240 | | | | | | 230×182-230×184 | |
| **ETL** | 7-19 | | 1 | | | | | | 1 | |
| **T2** |  | |  | | | | | |  | |
| **TR (ms)** | 3500-9186 | | 1090-6300 | | | | | | 1873-5000 | |
| **TE (ms)** | 77-128 | | 80-130 | | | | | | 70-100 | |
| **TI (ms)** | N/A | | N/A | | | | | | N/A | |
| **Section thickness (mm)** | 5 | | 5 | | | | | | 5-6 | |
| **Image slice spacing (mm)** | 1-2 | | 1.5-2 | | | | | | 1-1.5 | |
| **FA (°)** | 90-142 | | 90-150 | | | | | | 90 | |
| **number of averages/excitations** | 1-2 | | 1-2 | | | | | | 1-2 | |
| **Pixel size (mm^2^)** | 0.4297×0.4297-0.4688×0.4688 | | 0.3438×0.3438-0.8984×0.8984 | | | | | | 0.3993×0.3993-0.5324×0.5324 | |
| **Matrix** | 256×256-512×512 | | 256×256-448×448 | | | | | | 220×220-328×328 | |
| **FOV (mm^2^)** | 240×180-240×240 | | 220×184-240×240 | | | | | | 230×182-230×192 | |
| **ETL** | 24-32 | | 16-32 | | | | | | 12-17 | |
| **FLAIR** |  | |  | | | | | |  | |
| **TR (ms)** | 6100-9000 | | 4000-9000 | | | | | | 5000-6000 | |
| **TE (ms)** | 86-174 | | 79-127 | | | | | | 115-120 | |
| **TI (ms)** | 2000-2500 | | 1530-2500 | | | | | | 2000-2250 | |
| **Section thickness (mm)** | 5 | | 5-6 | | | | | | 5-6 | |
| **Image slice spacing (mm)** | 1-2 | | 1.5-2 | | | | | | 1-1.5 | |
| **FA (°)** | 90-160 | | 111-150 | | | | | | 90 | |
| **number of averages/excitations** | 1 | | 1-2 | | | | | | 1-2 | |
| **Pixel size (mm^2^)** | 0.4297×0.4297-0.4688×0.4688 | | 0.4297×0.4297-0.8984×0.8984 | | | | | | 0.4492×0.4492-0.8984×0.8984 | |
| **Matrix** | 256×224-256×256 | | 256×179-320×224 | | | | | | 176×176-256×256 | |
| **FOV (mm^2^)** | 240×180-240×240 | | 220×193-240×240 | | | | | | 230×182-230×206 | |
| **ETL** | 1-18 | | 16-23 | | | | | | 25-38 | |
| **DWI** |  | |  | | | | | |  | |
| **TR (ms)** | 4800-6000 | | 3400-6200 | | | | | | 2205-2798 | |
| **TE (ms)** | 74-88 | | 80-119 | | | | | | 51-90 | |
| **TI (ms)** | N/A | | N/A | | | | | | N/A | |
| **Section thickness (mm)** | 5 | | 5 | | | | | | 6 | |
| **Image slice spacing (mm)** | 1.5-2 | | 1.5-1.75 | | | | | | 1 | |
| **FA (°)** | 90 | | 90 | | | | | | 90 | |
| **number of averages/excitations** | 1 | | 1-3 | | | | | | 1 | |
| **Pixel size (mm^2^)** | 0.9375×0.9375 | | 0.5990×0.5990-1.5132×1.5132 | | | | | | 0.8984×0.8984-1.0268×1.0268 | |
| **Matrix** | 160×160 | | 152×152-220×220 | | | | | | 112×89-152×114 | |
| **FOV (mm^2^)** | 240×240 | | 220×220-240×240 | | | | | | 230×230 | |
| **ETL** | 1 | | 1-82 | | | | | | 47-59 | |
| **B value (s/mm^2^)** | 0, 1000 | | 0, 1000 | | | | | | 0, 1000 | |

**Supplementary Table S8.** Primers for RT-qPCR.

| PSMB8 (Forward): 5′-GGTCCTACATTAGTGCCTTACGG-3′ |
| --- |
| PSMB8 (Reverse): 5′-CGCAGATAGTACAGCCTGCATT-3′ |
| STUB1 (Forward): 5′-TCAAGGAGCAGGGCAATCGT-3′ |
| STUB1 (Reverse): 5′-CAGCGGGTTCCGGGTGAT-3′ |
| UBE2M (Forward): 5′-TGCGGATCCAGAAGGACATA-3′ |
| UBE2M (Reverse): 5′-GGTCGTCTGGATCTGAGAAGC-3′ |
| TGFBR1 (Forward): 5′-GACAACGTCAGGTTCTGGCTCAG-3′ |
| TGFBR1 (Reverse): 5′-TCCTCTCCAAACTTCTCCAAATCG-3′ |
| TGFBR2 (Forward): 5′-GTAGCTCTGATGAGTGCAATGAC-3′ |
| TGFBR2 (Reverse): 5′-CAGATATGGCAACTCCCAGTG-3′ |
| ITGB8 (Forward): 5′-CTGTTTGCAGTGGTCGAGGAGT-3′ |
| ITGB8 (Reverse): 5′-TGCCTGCTTCACACTCTCCATG-3′ |
| ITGAV (Forward): 5′-ATGCTCCATGTAGATCACAAGAT-3′ |
| ITGAV (Reverse): 5′-TTCCCAAAGTCCTTGCTGCT-3′ |
| SMAD2 (Forward): 5′-CGTCCATCTTGCCATTCACG-3′ |
| SMAD2 (Reverse): 5′-CTCAAGCTCATCTAATCGTCC-3′ |
| SMAD3 (Forward): 5′-GGAGAAATGGTGCGAGAAGG-3′ |
| SMAD3 (Reverse): 5′-GAAGGCGAACTCACACAGC-3′ |
| SMAD4 (Forward): 5′-GCATCGACAGAGACATACAG-3′ |
| SMAD4 (Reverse): 5′-CAACAGTAACAATAGGGCAG-3′ |
| Caspase-3 (Forward): 5′-CATGGAAGCGAATCAATGGACT-3′ |
| Caspase-3 (Reverse): 5′-CTGTACCAGACCGAGATGTCA-3′ |
| c-Myc(Forward): 5′-GTCAAGAGGCGAACACACAAC-3′ |
| c-Myc (Reverse): 5′-TTGGACGGACAGGATGTATGC-3′ |
| Cyclin D1 (Forward): 5′-GCTGCGAAGTGGAAACCATC-3′ |
| Cyclin D1 (Reverse): 5′-CCTCCTTCTGCACACATTTGAA-3′ |
| GAPDH (Forward): 5′-TGATGACATCAAGAAGGTGGTGAAG-3′ |
| GAPDH (Reverse): 5′-TCCTTGGAGGCCATGTGGGCCAT-3′ |

**Supplementary Figures with legends**


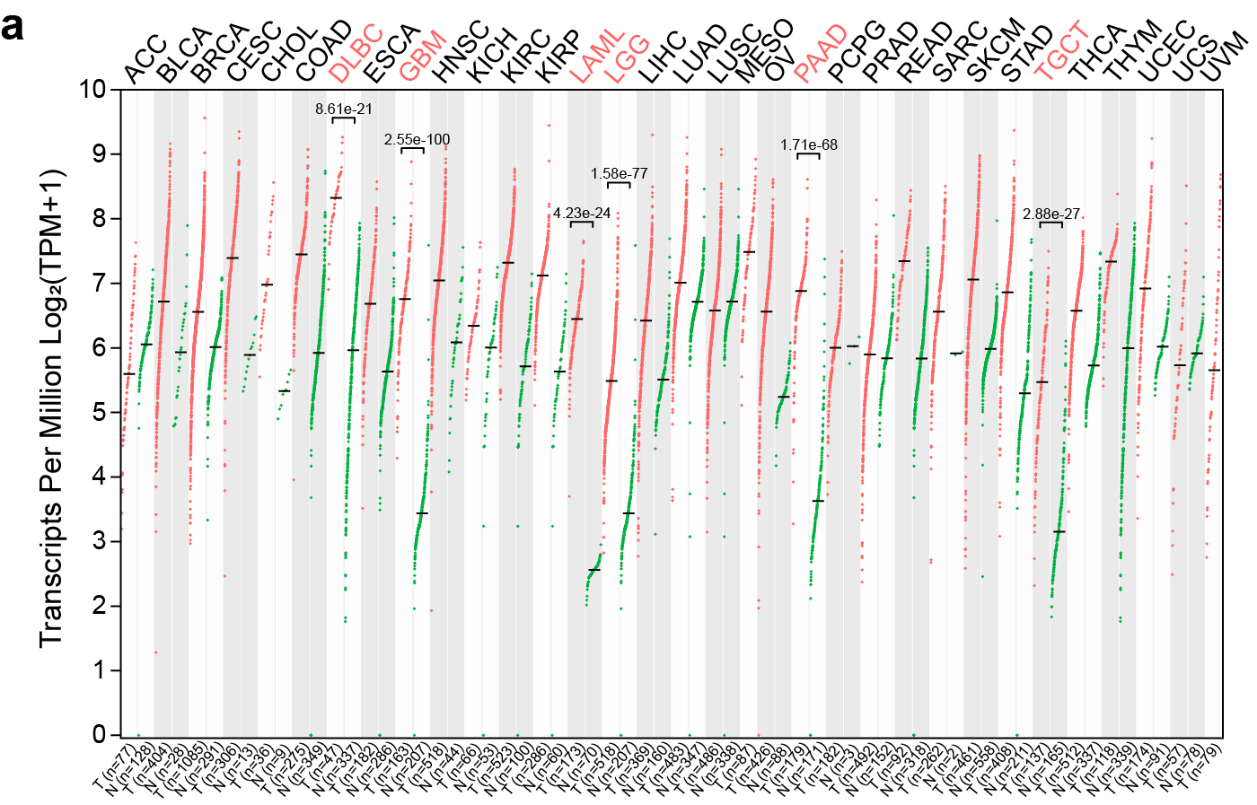
**Supplementary Figure S1.**

**Fig. S1. The expression of PSMB8 in 33 cancer types and matched TCGA normal data.**

**a** Expression of PSMB8 in 33 tumor samples (dot plot) using data from the Gene Expression Profiling Interactive Analysis database (http://gepia.cancer-pku.cn/). Each dot represents the expression of a sample.


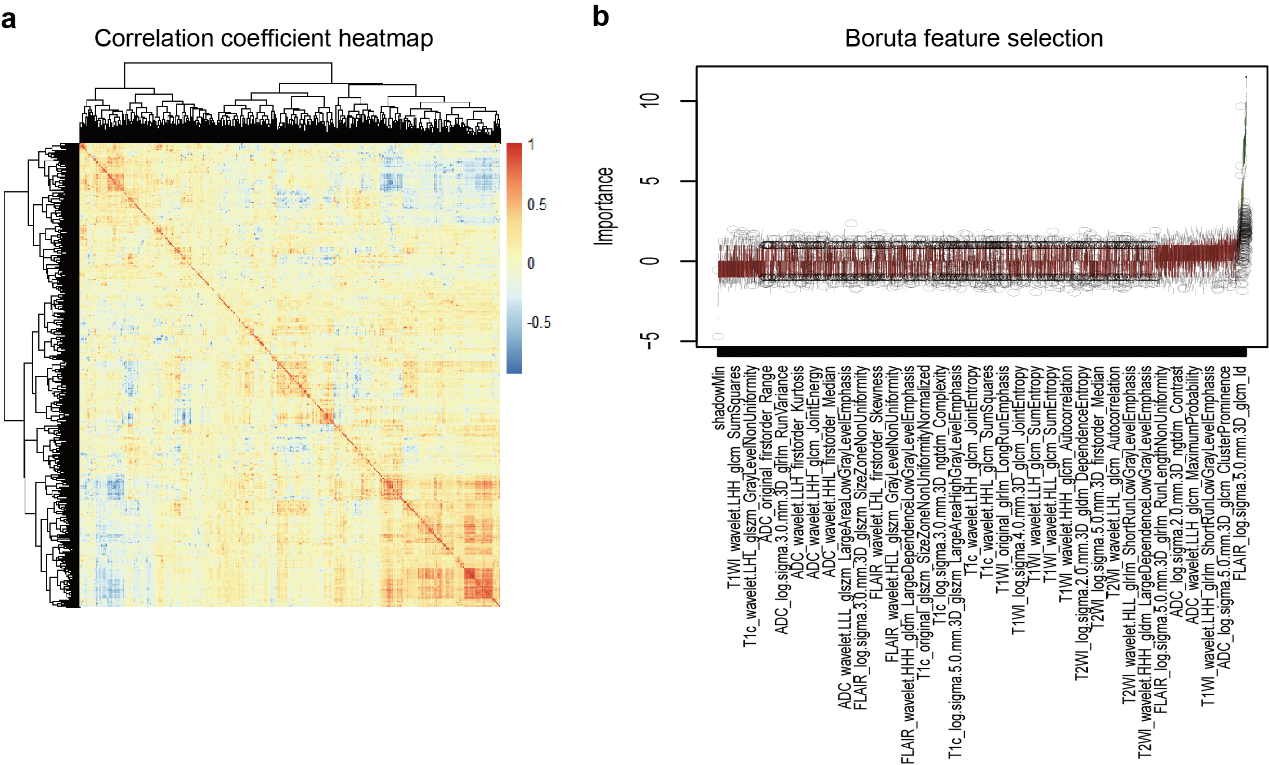
**Supplementary Figure S2.**

**Fig. S2. The heatmaps of the correlation coefficients of the imaging features and the result of the Boruta feature selection.**

**a** Correlation coefficient heatmap of the selected 1402 non-redundant features from multiparametric MRI. The dimmer the blue (lower) color, the red (higher) the correlation of a feature pair. The heatmaps indicated that the correlations between features were reduced after performing feature de-duplication. **b** The x-axis represented all features derived from the Caret algorithm in multiparametric MRI (T1, T2, FLAIR, T1c, ADC), and the y-axis represented the box plots of the feature importance values produced by the Boruta algorithm. Finally, 10 features with the largest importance values were selected from multiparametric MRI, as shown in green, whereas the discarded features with insignificant importance values were shown in yellow and red.


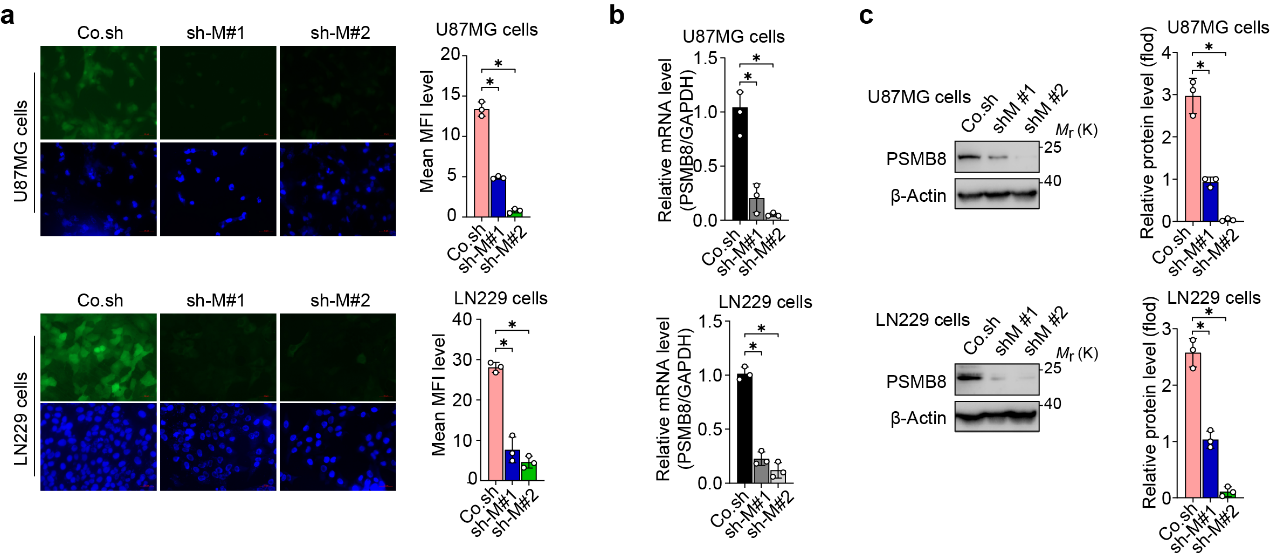
**Supplementary Figure S3.**

**Fig. S3. Knockdown the expression of PSMB8 in GBM cell lines.**

**a** Left: Representative fluorescence images of PSMB8 knockdown lentivirus infection in U87MG and LN229 GBM cells. Right: Mean fluorescence intensity (MFI) of PSMB8 knockdown lentivirus infection in U87MG and LN229 GBM cells; **b** qPCR analysis of PSMB8 in U87MG and LN229 cells transduced with shRNA against PSMB8 (sh-M #1 and sh-M #2) or a non-targeting control (Co.sh); **c** Representative IB and analysis of U87MG and LN229 GBM cells transduced with shRNA against PSMB8 (sh-PSMB8 #1 and sh-PSMB8 #2) or a non-targeting control (Co.sh). **p* < 0.05. Data are analyzed of three independent experiments and presented as mean ± SD (a right, b, and c right). Statistical analyses are performed using two-tailed Student’s *t*-test (a right, b, and c right).


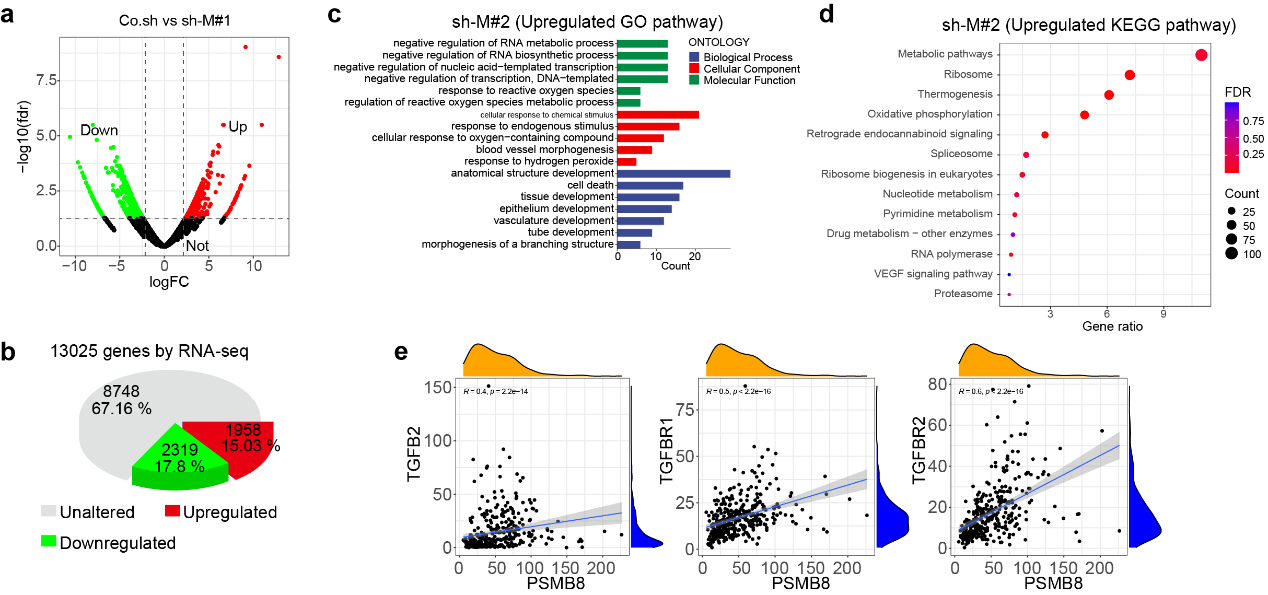
**Supplementary Figure S4.**

**Fig. S4. PSMB8 correlates with TGF-beta signaling pathway activity in glioma cancer.**

**a** A volcano plot illustrating differentially upregulated and downregulated genes of sh-M#2 compared to the Co.sh. Genes upregulated and downregulated are shown in red and green, respectively. **b** RNA-seq comparison of Co.sh and sh-M#1 revealed a total of 13025 genes, of which 1958 genes were upregulated and 2319 genes were downregulated. **c** Gene ontology functional clustering of genes that were upregulated for biological processes. **d** KEGG pathway analysis of genes that were upregulated targets between Co.sh and sh-M#1. **e** Positive correlation analysis of PSMB8 with TGFB2, TGFBR1, and TGFBR1 in our cohort of 333 glioma patients.
